# Histopathological and Molecular Profiling of Clear Cell Sarcoma and Correlation with Response to Crizotinib: An Exploratory Study Related to EORTC 90101 “CREATE” Trial

**DOI:** 10.3390/cancers13236057

**Published:** 2021-12-01

**Authors:** Che-Jui Lee, Elodie Modave, Bram Boeckx, Silvia Stacchiotti, Piotr Rutkowski, Jean-Yves Blay, Maria Debiec-Rychter, Raf Sciot, Diether Lambrechts, Agnieszka Wozniak, Patrick Schöffski

**Affiliations:** 1 Laboratory of Experimental Oncology, Department of Oncology, KU Leuven, 3000 Leuven, Belgium; jerry.lee@kuleuven.be (C.-J.L.); agnieszka.wozniak@kuleuven.be (A.W.); 2 VIB Center for Cancer Biology, VIB and Department of Human Genetics, KU Leuven, 3000 Leuven, Belgium; elodie.modave@kuleuven.be (E.M.); bram.boeckx@kuleuven.be (B.B.); diether.lambrechts@kuleuven.be (D.L.); 3 Department of Medical Oncology, Fondazione IRCCS Istituto Nazionale Tumori, 120133 Milano, Italy; silvia.stacchiotti@istitutotumori.mi.it; 4 Department of Soft Tissue/Bone Sarcoma and Melanoma, Maria Sklodowska-Curie National Research Institute of Oncology, 00001 Warsaw, Poland; piotr.rutkowski@pib-nio.pl; 5 Department of Medical Oncology, Centre Centre Léon Bérard & Université Claude Bernard Lyon I, 69008 Lyon, France; jean-yves.blay@lyon.unicancer.fr; 6 Department of Human Genetics, University Hospitals Leuven, KU Leuven, 3000 Leuven, Belgium; maria.debiec-rychter@kuleuven.be; 7 Department of Pathology, University Hospitals Leuven, KU Leuven, 3000 Leuven, Belgium; raf.sciot@uzleuven.be; 8 Department of General Medical Oncology, Leuven Cancer Institute, University Hospitals Leuven, 3000 Leuven, Belgium

**Keywords:** clear cell sarcoma, molecular analysis, CREATE, crizotinib

## Abstract

**Simple Summary:**

Clear cell sarcoma (CCSA) is a rare subtype of soft tissue sarcoma characterized by *EWSR1* rearrangement and subsequent MET upregulation. The European Organisation for Research and Treatment of Cancer 90101 phase II trial evaluated the MET inhibitor crizotinib in CCSA but resulted in only sporadic responses. The aim of this exploratory study was to identify the molecular alterations potentially relevant for the treatment outcome by using archival CCSA samples and trial-related clinical data. We characterized MET signaling and revealed an infrequent activation of MET, which may explain the lack of response to crizotinib in the disease cohort. Based on sequencing analyses, we discovered copy number alterations, mutations and dysregulated pathways with potentially predictive or prognostic values for patients’ outcomes. This work describes the molecular heterogeneity in CCSA and provides deep insight into the biology of this ultra-rare malignancy, which may potentially lead to better therapeutic approaches.

**Abstract:**

Clear cell sarcoma (CCSA) is characterized by a chromosomal translocation leading to *EWSR1* rearrangement, resulting in aberrant transcription of multiple genes, including *MET*. The EORTC 90101 phase II trial evaluated the MET inhibitor crizotinib in CCSA but resulted in only sporadic responses. We performed an in-depth histopathological and molecular analysis of archival CCSA samples to identify alterations potentially relevant for the treatment outcome. Immunohistochemical characterization of MET signaling was performed using a tissue microarray constructed from 32 CCSA cases. The DNA from 24 available tumor specimens was analyzed by low-coverage whole-genome sequencing and whole-exome sequencing for the detection of recurrent copy number alterations (CNAs) and mutations. A pathway enrichment analysis was performed to identify the pathways relevant for CCSA tumorigenesis. Kaplan–Meier estimates and Fisher’s exact test were used to correlate the molecular findings with the clinical features related to crizotinib treatment, aiming to assess a potential association with the outcomes. The histopathological analysis showed the absence of a MET ligand and MET activation, with the presence of MET itself in most of cases. However, the expression/activation of MET downstream molecules was frequently observed, suggesting the role of other receptors in CCSA signal transduction. Using sequencing, we detected a number of CNAs at the chromosomal arm and region levels. The most common alteration was a gain of 8q24.21, observed in 83% of the cases. The loss of chromosomes 9q and 12q24 was associated with shorter survival. Based on exome sequencing, 40 cancer-associated genes were found to be mutated in more than one sample, with *SRGAP3* and *KMT2D* as the most common alterations (each in four cases). The mutated genes encoded proteins were mainly involved in receptor tyrosine kinase signaling, polymerase-II transcription, DNA damage repair, SUMOylation and chromatin organization. Disruption in chromatin organization was correlated with longer progression-free survival in patients receiving crizotinib. Conclusions: The infrequent activation of MET may explain the lack of response to crizotinib observed in the majority of cases in the clinical trial. Our work describes the molecular heterogeneity in CCSA and provides further insight into the biology of this ultra-rare malignancy, which may potentially lead to better therapeutic approaches for CCSA.

## 1. Introduction

Clear cell sarcoma (CCSA) is an ultra-rare subtype of soft tissue sarcoma (STS) that accounts for approximately 1% of all STS cases [1,2]. This disease has a strong tendency toward local recurrence and diffuse distant metastasis and is characterized by a profound resistance to chemo- and radiotherapy [3,4,5]. CCSA is often diagnosed in the extremities but can occur in any part of the body [6]. Approximately 90% of CCSA harbor a t(12; 22)(q13; q12) translocation causing an Ewing sarcoma breakpoint region 1/activating transcription factor-1 (*EWSR1*/*ATF1*) rearrangement, resulting in the aberrant activation of microphthalmia-associated transcription factor (MITF) [7,8]. MITF activates the transcription of multiple genes, including *MET*, and the subsequent overexpression of the receptor tyrosine kinase (RTK) MET, which is suggested as a potential target for CCSA treatment [9,10].

A prospective phase II trial, European Organization for Research Treatment of Cancer (EORTC) 90101 “CREATE”, evaluated the oral MET/ALK/ROS1 inhibitor crizotinib in patients with CCSA [11]. The activity of this agent was assessed in MET-positive and MET-negative cases, where the MET status was defined by fluorescence in situ hybridization (FISH), detecting *EWSR1* rearrangements in at least 15% of the tumor cells. This trial demonstrated that crizotinib provided some clinical benefits in patients with locally advanced or metastatic CCSA but did not meet its primary endpoint (overall response rate), as only one partial response was observed among 26 evaluable patients. This suggests that other factors may contribute to the oncogenesis and progression of CCSA in addition to MET upregulation.

It has been reported that *EWSR1* rearrangement is required for MET expression and that MET inhibition could decrease CCSA cell growth in vitro [10]. The molecular epidemiology of the MET status (expression and activation) is poorly described in the literature. The associated molecules involved in MET signaling, such as the MET ligand hepatocyte growth factor (HGF), can possibly have an impact on the function of MET in CCSA. Additionally, other molecular event can possibly influence the response to crizotinib, including *MET* amplification or mutations, and bypass the signaling pathways [12]. MET-addicted non-small cell lung cancer (NSCLC) with high-level *MET* amplification and *MET* exon 14 alterations is known to be sensitive to treatment with crizotinib, while the presence of secondary *MET* mutations is described as a potential resistance mechanism [13,14]. Similar phenomena may play a role in the sensitivity of CCSA to crizotinib.

We performed an in-depth histopathological and molecular analysis of archival CCSA tumor materials from patients who participated in the CREATE trial, based on tissue microarrays (TMA) constructed from leftover tissue. Using immunohistochemistry and high-throughput sequencing, we characterized the relevant molecules in the MET-signaling pathway and assessed the copy number changes and mutational profiles, with the aim to identify potentially predictive/prognostic biomarkers and novel therapeutic targets for this rare cancer. 

## 2. Material and Methods

### 2.1. Immunohistochemical Characterization of the MET-Signaling Pathway

A total of 36 formalin-fixed and paraffin-embedded (FFPE) tissue samples from patients with CCSA were available and had been archived in a central biorepository (BioRep, Milan, Italy). The diagnosis of CCSA was centrally confirmed as part of the clinical trial. Thirty-two of the tumor blocks were used to construct a TMA with 1.5-mm triplicate cores per case [15]. A series of TMA sections were used for immunohistochemical characterization of the MET pathway-related molecules [16]. Commercially available antibodies against MET, GRB2-associated-binding protein 1 (GAB1), mitogen-activated protein kinase (MAPK), protein kinase B (AKT), ribosomal S6 kinase (S6) and their phosphorylated (activated) forms, as well as MITF and HGF, were used for immunohistochemistry (IHC). Optimized conditions and source of the antibody are listed in Table S1. Stained TMA slides were subsequently scanned and evaluated blindly by an investigator (CJL) using an Olympus BX43 microscope and cellSens software (Olympus, Tokyo, Japan). The analysis was done according to the scoring intensity: negative, weakly positive, moderately positive and strongly positive. Cores with more than 80% of the tissue section absent after processing were considered unevaluable. For cases with more than one available core on the TMA, the mean of the scoring was recorded as the final result.

### 2.2. Low-Coverage Whole-Genome Sequencing

DNA was extracted from 24 archived FFPE tumor samples having a quality acceptable for libraries preparation. Illumina^®^ HiSeq4000 (Illumina, San Diego, CA, USA) was used for sequencing at a low coverage (±0.1×). Raw sequencing reads (50 bp) were mapped to the human reference genome (GRCh37/hg19 version) using Burrows-Wheeler Aligner (BWA v0.5.8 a, Massachusetts Institute of Technology, Cambridge, MA, USA) and sorted with SAMtools (v0.1.19, Massachusetts Institute of Technology, Cambridge, MA, USA). Picard tools were used to remove duplicates. QDNASeq and ASCAT were used to count and segment the aligned reads in bins of 50 kb [17,18]. The Genomic Identification of Significant Targets in Cancer algorithm (GISTIC, Broad Institute, Cambridge, MA, USA) was used to identify the most frequent and significant chromosomal alterations. A region was considered deleted if the log value was <−0.1, while it was amplified if the log value was >0.1. The Benjamini–Hochberg method was used to correct for multiple testing, and significant CNAs with a cut off *q*-value < 0.25 were selected [19]. CNAs were defined as a broad (arm level) event if the alterations were spanning >75% of a chromosomal arm, while alterations spanning <75% were considered as focal CNAs (region level).

### 2.3. Whole-Exome Sequencing

Libraries prepared for low-coverage whole-genome sequencing were enriched for exomic sequences using the SeqCapV3 exome enrichment kit (Roche, Basel, Switzerland) following the manufacturer’s instructions. They were sequenced on HiSeq4000 using a flow cell, resulting in 2 × 150-bp end reads that were further mapped and sorted, and the duplicates were removed as described above. Next, base recalibration, local realignment and single-nucleotide variant calling were performed with the Genome Analysis Tool Kit (GATK, Broad Institute, Cambridge, MA, USA). Dindel was used for calling small insertions and deletions (indels). Mutations with a coverage <10× or a quality score <30 for substitutions or <50 for indels were discarded. Since no germline samples were available, a strict filtering strategy was applied based on publicly available databases to exclude the common single-nucleotide polymorphisms. Mutations occurring in large databases (ESP, 1 kg, ExAC) with an allelic frequency >0.001, as well as mutations occurring in smaller, appropriate databases (bitsTrio, inhouseDB, cg69 and GoNL), were removed if they occurred in more than one individual. The Cancer Gene Consensus (CGC) set, developed by the Catalogue of Somatic Mutations in Cancer databases (COSMIC v89, Wellcome Trust Sanger Institute, Cambridge, UK), was applied for a further analysis to include genes that have been implied in cancer [20]. We also used PolyPhen-2 (Harvard University, Cambridge, MA, USA) to predict the possible impacts of amino acid substitutions on the structures and functions of protein products [21], and this analysis was supplemented by VarSome (Saphetor, Lausanne, Switzerland) if the mutations were not analyzable by PolyPhen-2 [22]. Visualization of the mutations was performed using MutationMapper (cBioPortal, Memorial Sloan Kettering Cancer Center, New York, NY, USA) [23]. To assess the clinical applications of the mutated genes, we used the Drug Gene Interaction Database (DGIdb, Washington University School of Medicine, St. Louis, MO, USA) to predict the potential druggability [24].

### 2.4. Pathway Enrichment Analysis

To identify the pathways that were significantly dysregulated in CCSA, we conducted a pathway enrichment analysis using g:Profiler (University of Tartu, Tartu, Estonia) to identify disrupted pathways enriched in the list of mutated CGC genes that were detected by WES. The g:Profiler is a tool combining bioinformatic and statistic methods to pick up pathways where the genes present in the dataset are significantly enriched or overrepresented, as compared to all the genes in the genome [25]. All pathways were tested for enrichment in the gene list based on Reactome (European Molecular Biology Laboratory, European Bioinformatics Institute, Hinxton, UK), one of most common databases for the investigation of molecular pathways [26]. Pathways with minimum sizes of 5 genes per set were considered for the analysis. The significance was computed using Fisher’s exact test and multiple test correction. Pathways with *q*-values < 0.01 were considered significant. Visualization was done by using EnrichmentMAP, AutoAnnotate and the Markov Cluster algorithm (Bader Lab, University of Toronto, Toronto, ON, Canada) in a Cytoscape v3.7.2 (Institute of Systems Biology, Seattle, WA, USA) environment [27,28].

### 2.5. Clinical Outcome and Statistical Analysis

The response to crizotinib was evaluated using Response Evaluation Criteria in Solid Tumors (RECIST v1.1), as previously reported [11]. A total of 26 patients were assessable for the primary endpoint: an objective response, according to RECIST v1.1 [29]. For the purpose of the current translational project, patients with at least a stable disease (SD) as the best response were defined as having achieved disease control, while patients with a RECIST progressive disease (PD) were defined as non-responders. Progression-free survival (PFS) and overall survival (OS) were available as secondary endpoints from the clinical trial. Fisher’s exact test was used to test whether the response groups were significantly enriched for certain alterations. Kaplan–Meier estimates with the log-rank test were used to assess the correlation between the molecular findings and patient survival. For the correlation between survival and the expression of MET-related molecules, the tumors from the patients were grouped as having low (negative or weakly positive) or high expression (moderately or strongly positive) based on the intensity of the IHC staining. A statistical analysis was performed using GraphPad Prism v7 (GraphPad, San Diego, CA, USA) and SPSS v27 (IBM, Armonk, NY, USA). *p* values < 0.05 were considered significant.

## 3. Results

### 3.1. Patient Cohort

Archival tumor material was available from 34 out of 36 CCSA patients enrolled in EORTC 90101. These samples included 30 MET-positive tumors according to the protocols, three MET-negative and one unevaluable for MET status. Among 26 out of the 34 eligible patients in the trial, only one achieved a partial response (PR), and 17 had SD as the best response to crizotinib by RECIST 1.1. PD was the best response in 8 cases. Six patients were treated but did not reach the primary end point, and two cases were not treated [11]. The biological materials obtained from 34 cases included 21 primary tumors, 12 metastatic lesions and one unknown disease status. The male-to-female ratio in this subset was 1.8, and the median age at diagnosis was 41 years (range 17–62). The median OS and PFS were 8.3 and 3.5 months, respectively. Among the 34 available samples, 32 were included in the TMA, and 24 underwent sequencing. The clinicopathological variables for each case are summarized in Table 1.

### 3.2. Immunohistochemical Characterization of MET Signaling

**Figure 1:**
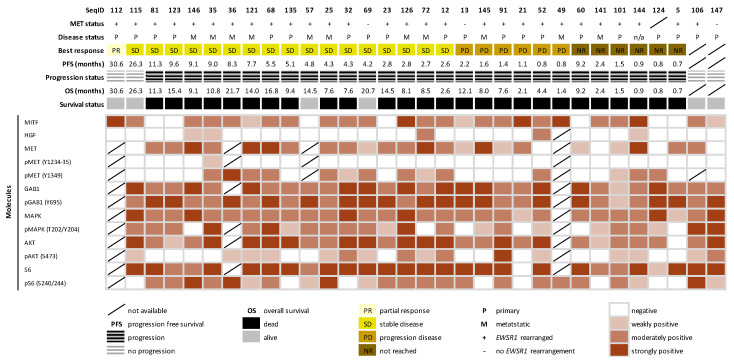
Overview and summary for immunohistochemical characterization of the MET-signaling pathway in clear cell sarcoma. The heatmap presents an overview for the expression profile of MET pathway-related molecules in 32 cases. The expression level was determined by the mean of the staining intensity among the cores per case.

To explore the expression and activation of MET and related molecules involved in RTK signaling, we performed IHC using TMA constructed from 32 cases. A series of TMA slides were successfully stained with antibodies against MITF, HGF, (p)MET, (p)GAB1, (p)MAPK, (p)AKT and (p)S6, with evaluable rates from 88% to 100% per staining. MITF expression was observed in 78% of the samples, including three MET-negative cases, while expression for HGF and MET was found in 16% and 82% of the samples, respectively. To assess the activation of the MET receptor, two phosphorylated MET sites (Tyr1234 to 1235 and Tyr1349) were evaluated, showing immunopositivity in 4% and 50% of the cases, respectively. The downstream-signaling molecules and their phosphorylated forms were positive in most of the cases, apart from phosphorylated AKT i.e., GAB1 (100%), pGAB1 (100%), MAPK (100%), pMAPK (79%), AKT (97%), pAKT (53%), S6 (97%) and pS6 (74%). Notably, 20%, 43% and 24% of the cases expressing MAPK, AKT and S6 were absent for the phosphorylated form, suggesting a dominance of MAPK and S6 activation in CCSA. The immunoreactivity for each molecule is summarized in Figure 1 and Table S2. Representative examples of the immunohistochemical analysis of MET-related molecules can be found in Figure S1.

### 3.3. Global CNA Profile in CCSA

**Figure 2:**
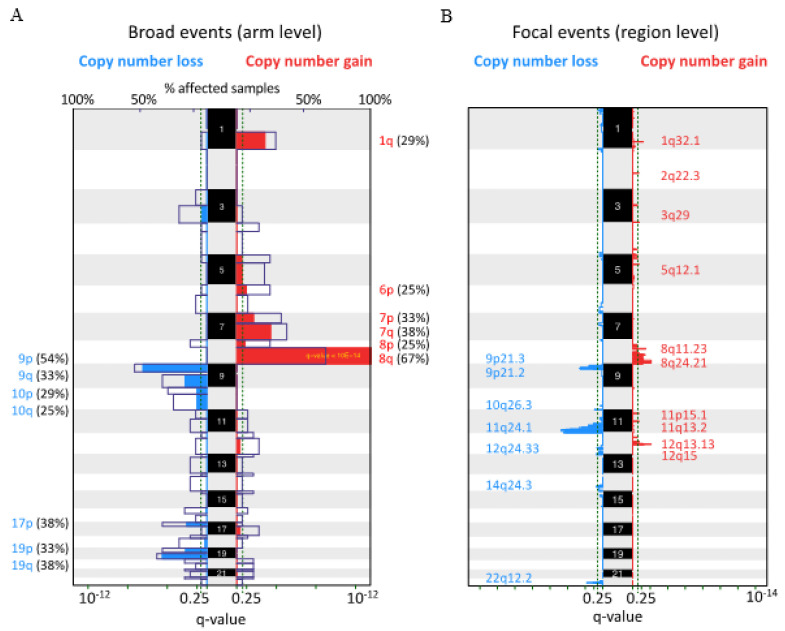
Global copy number alteration profile in clear cell sarcoma. The recurrent alterations were identified at the (**A**) broad and (**B**) focal levels in 24 cases. Colored peaks represent gains/losses by broad (chromosome arm) or focal (region) events; the threshold of significance is a *q*-value < 0.25; numbers in parentheses represent the % of samples affected by copy number alterations.

To reveal the CNA profile in CCSA, we performed low-coverage whole-genome sequencing and a GISTIC analysis allowing the detection of recurrent regions affected by CNAs in 24 assessable samples. A number of the chromosomal arm-level (broad events) and regional-level (focal events) regions affected by CNAs were detected. The most frequent arm-level CNA was a gain of chromosome 8q, which was present in 16 out of 24 (67%) cases. Other broad events detected were the gain of 7q (38%), 7p (33%), 1q (29%), 6p (25%) and 8p (25%), with frequent losses of 9p (54%), 19q (38%), 9q (33%), 17q (33%), 19q (33%), 10p (29%) and 10p (25%) (Figure 2A). Furthermore, focal CNAs were detected at 17 loci (10 regions gained and seven deleted). An overrepresented focal copy number gain was observed at 8q24.21 (83%), followed by 8q11.23 (67%), 1q32.1 (42%), 12q15 (38%), 5q12.1 (29%), 12q13.13 (25%), 3q29 (17%), 11p15.1 (17%), 11q13.1 (17%) and 2q22.3 (8%). The recurrent losses were at 9p21.3 (63%), 9p21.2 (63%), 10q26.3 (63%), 11q24.1 (42%), 12q24.33 (29%), 22q12.2 (29%) and 14q24.3 (25%) (Figure 2B). The CGC genes involving regions affected by focal CNAs are listed in Table 2.

### 3.4. Mutational Landscape of CCSA

**Figure 3:**
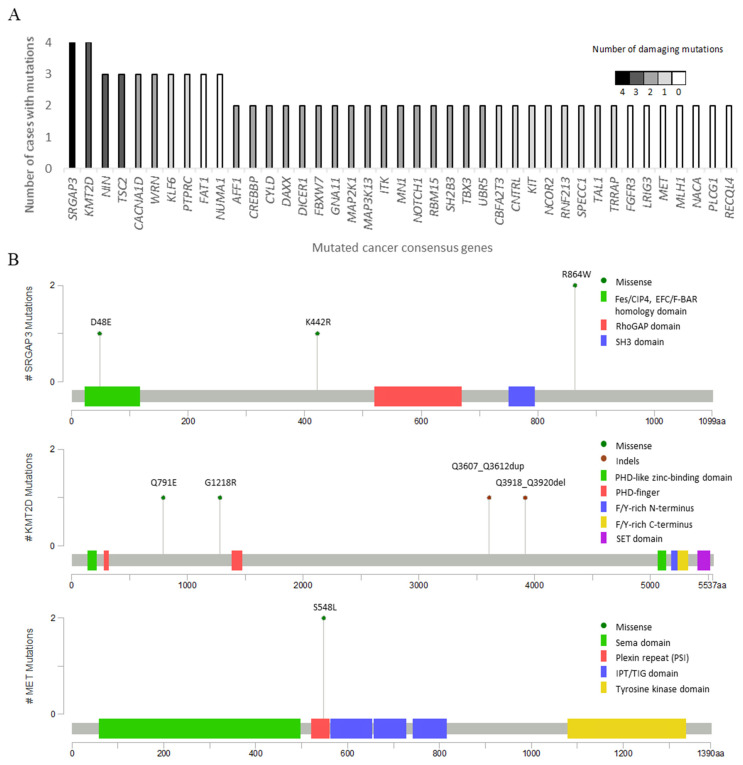
Mutational profile in clear cell sarcoma with recurrent and damaging mutations identified in 24 cases. (**A**) Cancer gene consensus-associated genes altered by nonsynonymous mutations in at least two out of 24 clear cell sarcoma cases. The *y*-axis represents the number of cases with nonsynonymous mutations, and the *x*-axis represents mutated genes. The gray-scale-colored column serves as the number of cases with damaging mutations. (**B**) The lollipop plots map the mutations in *SRGAP3*, *KMT2D* and *MET* on a linear protein sequence and their domains (colored boxes). The *y*-axis represents the number of cases with mutations, and the *x*-axis represents an amino acid sequence of mutated genes. The colored codes of the mutation diagram circles represent different mutation types (green: missense and brown: indels).

To assess the mutational landscape of CCSA, the DNA used for the CNA analysis was also subjected to whole-exome sequencing. We sequenced them at an average coverage of 77.2× and detected a total of 6181 mutations with an average of 258 mutations per sample (range 156–473). Among all the alterations detected, 4201 were no-synonymous mutations, including 3548 missense, 134 nonsense mutations and 519 insertions and deletions (indels). For further analysis, we focused on the genes that were previously documented in the CGC set. A total of 105 CGC genes affected by 211 mutations, with the average of nine (range 3–22) per case, were identified, and 40 of them were found to be mutated in more than one case. Only 22 altered genes (*SRGAP3*, *KMT2D*, *NIN*, *TSC2*, *CACNA1D*, *WRN*, *AFF1*, *CREBBP*, *CYLD*, *DAXX*, *DICER1*, *FBXW7*, *GNA11*, *MAP2K1*, *MAP3K13*, *ITK*, *MN1*, *NOTCH1*, *RBM15*, *SH2B3*, *TBX3* and *UBR5*) displayed damaging mutations (PolyPhen-2 or VarSome) in more than one case. The proportion of mutations, including those with damaging phenotypes, is presented in Figure 3A. In the analyzed cohort, the mutations in *SRGAP3* and *KMT2D* were the most common alterations (in four cases). *SRGAP3* encodes a product inhibiting Rho GTPase Rac1 and interacts with actin remodeling proteins to regulate the cytoskeleton [30]. *KMT2D* plays a critical role in epigenetic regulation and may have an impact on development, differentiation, metabolism and tumor suppression [31]. We also detected two cases with *MET* mutations (p.S548L) located in the plexin–semaphorin–integrin (PSI) domain. However, this mutation was predicted to be non-pathognomonic. Figure 3B presents the positions and types of mutations in *SRGAP3, KMT2D* and *MET*. Among the genes mutated in more than one case, we identified 16 potentially druggable genes (*CACNA1D, PTPRC*, *FAT1, CREBBP*, *MAP2K1*, *MAP3K13*, *ITK*, *NOTCH1*, *KIT*, *NCOR2*, *TRRAP*, *FGFR3*, *LRIG3*, *MET*, *MLH1* and *PLCG1*) using DGIdb.

### 3.5. Gene Alteration Landscape and Pathway Enrichment Analysis in CCSA

**Figure 4:**
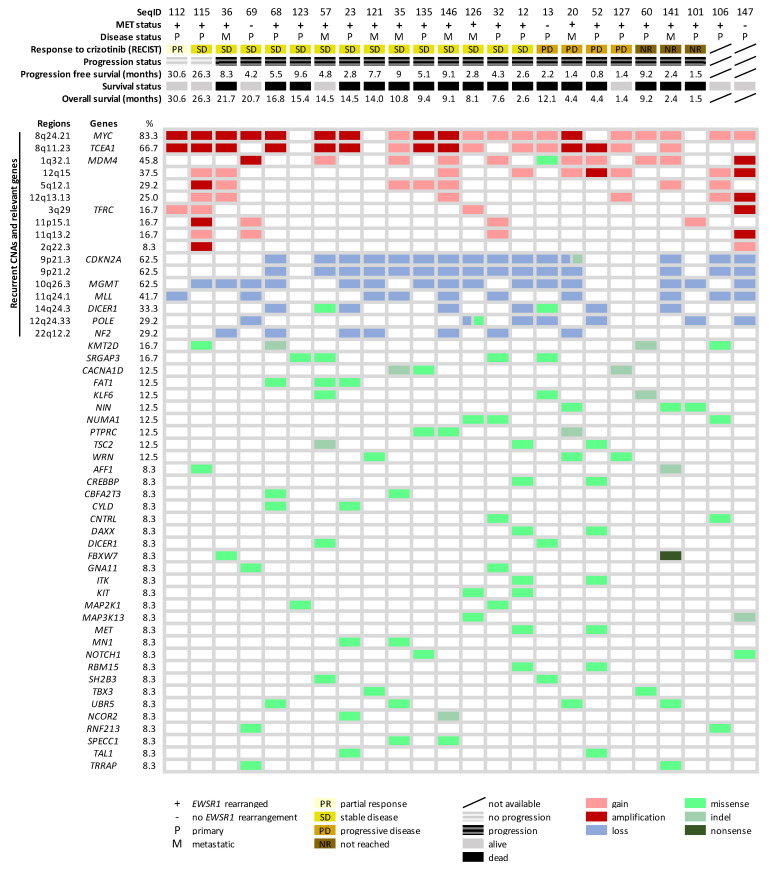
An overview of the genetic alteration landscape and crizotinib-related clinical data in 24 clear cell sarcomas. On the top, the clinical data of each patient is listed, ordered based on their response to crizotinib. Genomic regions, as well as genes (cancer consensus gene-associated and common cancer susceptibility genes) affected by recurrent copy number alterations and mutations, are ranked according to the frequency.

**Figure 5:**
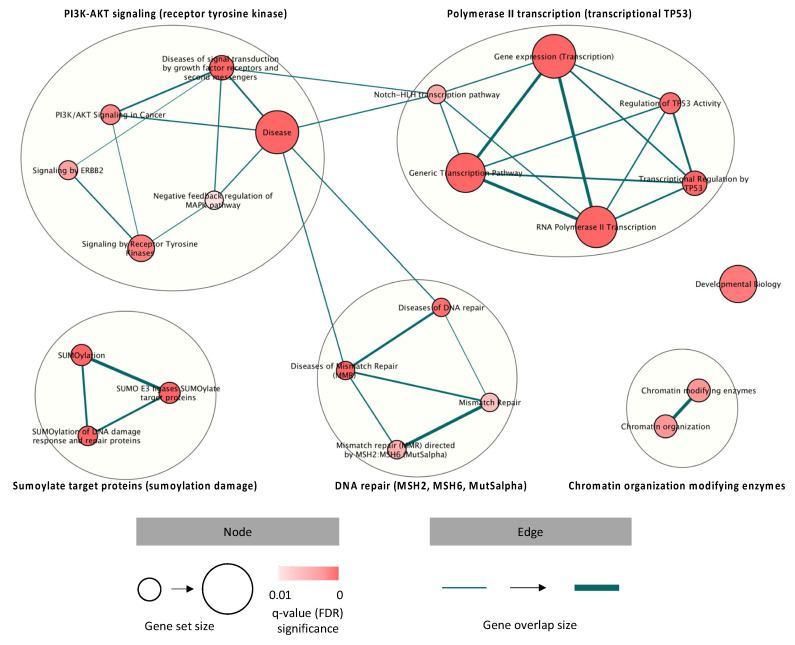
Pathways disrupted by mutations in the CGC set, identified in clear cell sarcoma, revealing dysregulations in receptor tyrosine kinase signaling, polymerase II transcription, DNA damage and mismatch repair, SUMOylation damage and chromatin organization-modifying enzymes. Red-coded nodes represent the significantly dysregulated pathways, and the significance is determined by the intensity of the color. The size of the node indicates how many genes are documented in each pathway. The edges represent the associations between the pathways, and the thickness is used to present the associated level.

Next, we combined the molecular findings in this study cohort, aiming to explore the disease biology beyond *EWSR1* rearrangement. An overview of the molecular alterations detected in the analyzed cohort is summarized in Figure 4. Furthermore, we performed a pathway enrichment analysis to allocate the mutated CGC genes to predefined pathways (Reactome), focusing on the identification of significant dysregulations at the pathway level. Subsequently, five clusters involving 22 significantly disrupted pathways were identified, including PI3K-AKT signaling; polymerase II transcription; DNA damage and mismatch repair (MMR); SUMOylate target proteins (reversible posttranslational modification by small ubiquitin-like modifiers) and chromatin organization-modifying enzymes (histone modification, DNA modification and transcription) (Figure 5). The overrepresented terms of the disrupted pathways are listed in Table S3. To better understand how MET signaling was dysregulated, we compared the expression profiles of the activated molecules involved in MET signaling with genomic alterations in the RTK-related pathways detected in CCSA. We found that the RTK-, ERBB2- and GFR-signaling pathways were altered in 13, 6 and 14 out of 21 comparable cases, and only one case presented activated MAPK and S6 in the absence of activated MET or genomic alterations in these signaling pathways (Figure S2).

### 3.6. Association between Molecular Findings and Clinical Features Related to Crizotinib Treatment

**Figure 6:**
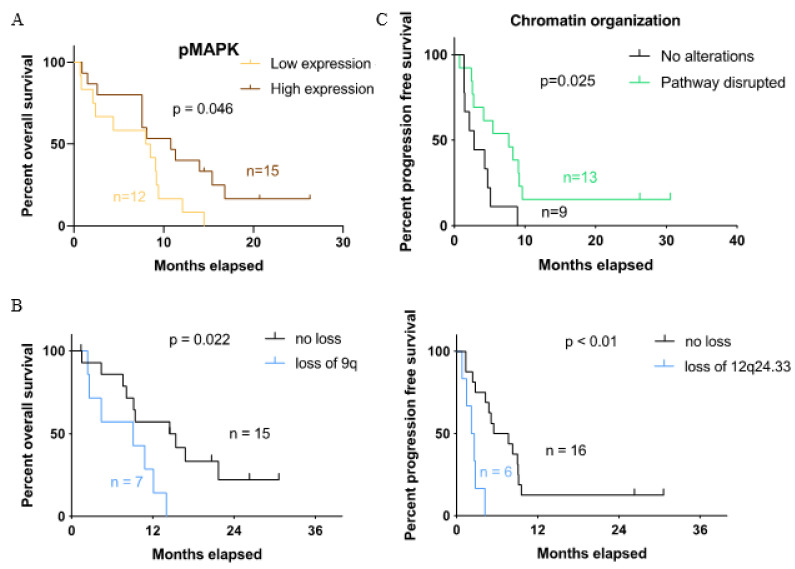
Survival curves for the correlation between the molecular findings and clinical features related to crizotinib treatment. (**A**) Kaplan–Meier estimates for different levels of immunoreactivity divided by low (negative and weakly positive) and high (moderately and strongly positive) expressions. Overall survival is compared between high and low expressions of phosphorylated MAPK. (**B**) Overall survival and progression-free survival for cases with different copy number alteration statuses of chromosomes 9q and 12q24.33. (**C**) Progression-free survival for cases with and without alterations in chromatin organization.

To identify the potential prognostic factors, we correlated the molecular findings with the clinical parameters, including the disease status, OS, PFS and response to crizotinib. In the immunohistochemical analysis, the Kaplan–Meier estimates showed that the activation of MAPK was associated with longer OS (*p* = 0.046) (Figure 6A). In the genomic analysis, the loss of chromosomes 9q and 12q24.33 was associated with shorter OS (*p* = 0.02) and PFS (*p* < 0.01), respectively (Figure 6B). We also observed more often copy number gains of chromosomes 1q (5/8 vs. 2/16, *p* = 0.02), 7p (6/8 vs. 2/16, *p* = 0.005) and 7q (7/8 vs. 2/16, *p* < 0.001) in metastatic lesions compared to the primary tumors, though they were not associated with patient survival. Figure 6C demonstrates the association between chromatin organization deficiency (disrupted pathways involving histone modifications, DNA modifications and transcriptions) with prolonged PFS (*p* = 0.025). We tried to perform a more advanced statistical analysis to support our oberservation. However, we were not able to obtain conclusive results due to the disease cohort being of a small sample size and with limited clinical data. 

## 4. Discussion

CCSA is known as a translocation-associated sarcoma that is associated with *EWSR1*-rearrangements, leading to aberrant MET expression [32]. Crizotinib is a MET inhibitor and was therefore tested in a prospective phase II trial, EORTC 90101 CREATE, in patients with this ultra-rare entity. The results from this trial suggested that MET may not be the most relevant or only target for therapy in CCSA [11]. Little is known about the biology of this disease. Therefore, we aimed to characterize the molecular alterations in CCSA using archival tumor samples (*n* = 34) collected in the context of the clinical trial. The recurrent alterations were further analyzed and correlated with the treatment outcome, with the aim of identifying the therapeutic targets and potential predictive/prognostic biomarkers. The results of this work were a comprehensive analysis of the genomic landscape of CCSA including an in-depth analysis of MET signaling in this rare malignancy.

*EWSR1*–*ATF1* fusion has been proven to upregulate its transcriptional target of the MITF melanocytic transcription factor, leading to subsequent MET overexpression in CCSA [8]. Since a MET inhibitor crizotinib was tested in the “CREATE” trial, we were interested in the role of the MITF-MET-signaling pathway in the crizotinib response. Therefore, we characterized the MITF-MET-signaling pathway in 32 CCSA cases. Interestingly, two cases without *EWSR1* rearrangement showed MITF expression, while no MITF expression was detected by IHC in seven specimens with altered *EWSR1*, suggesting that not all *EWSR1*-rearanged cases induce MITF overexpression. As previously reported, CCSA with *EWSR1-CREB1* rearrangement had little or no melanocytic signatures (absence of MITF) compared to those with common fusion involving *ATF1* [33]. It might explain the counterintuitive observation in our cohort, and we cannot exclude other fusion partners in our subset of CCSA, which should be confirmed with additional analyses. Next, MET expression was seen in three cases without MITF expression, while three cases presenting MITF had no MET expression. This suggests that MET upregulation could be MITF-independent in some cases. For instance, MET upregulation can also result from other mechanisms, such as AP1-enhancing transcription and eIF-mediated translation [34]. 

Under physiological conditions, MET activation requires the presence of its ligand HGF. In our study, a relatively low frequency of HGF expression (16%, weak or moderate positivity) and MET phosphorylation at Tyr1234–35 (tyrosine kinase domain, 4%) was observed, while 50% of the cases were positive for MET phosphorylation at Tyr1349 (C-terminal). At the same time, the downstream signaling pathway was found activated in a majority of the cases, as illustrated by frequent and strong positivity for pGAB1 (97%) and pMAPK (79%). These findings suggested the presence of other activating mechanisms in CCSA. Several RTKs (e.g., EGFR, RON and others) engaged with their ligands could transactivate MET and the subsequent signaling in an HGF-independent manner [34,35,36], which may explain the cases with MET Tyr1349 phosphorylation in the absence of HGF. Among these membrane molecules, the association between EGFR and MET has been studied in more detail. Activated EGFR was reported to transactivate MET undergoing subsequent signaling and/or directly transduce the MAPK-signaling pathway via GAB1 [36,37]. Therefore, the role of EGFR in CCSA should be further explored. Nevertheless, a high frequency of S6 activation (74%) was observed in our cohort, suggesting mTOR activation, which is also supported by a previous research article [38]. That may indicate the potential utility of mTOR inhibitors in CCSA. Our comprehensive characterization of MET signaling provided information that can be useful for the development of novel treatment strategies; however, we did not find clear associations with the outcome on crizotinib.

Using low-coverage whole-genome sequencing, we detected recurrent CNAs in 24 CCSA samples, where a gain of chromosome 8q (67%) was the most common broad alteration. Similarly, a number of individual cases harboring gains of chromosome 8q were found in previous studies [7,39], indicating that this alteration and the genes located in this region might be important oncogenic drivers for the development of CCSA. Furthermore, the most commonly affected regions were a gain of 8q24.21 (83%) and loss of 9p21.3 (63%), where *MYC* and *CDKN2A* are located, respectively. Ozenberger et al. recently reported *MYC* amplifications and *CDKN2A* deletions as recurrent CNA in 13 CCSA cases and demonstrated that MYC overexpression contributes to tumorigenesis in this disease [40]. Aberrant *MYC* (gain) and *CDKN2A* (loss or mutation) have been frequently detected in various human cancers, including melanoma [41,42,43]. Interestingly, CCSA was previously (mis)classified as a “malignant melanoma of soft parts”, because it shares similar clinicopathological and molecular profiles with melanoma [3,44,45]. It is possible that aberrant MYC also contributes to CCSA sarcomagenesis and could be a selective target for this entity. A recent study has demonstrated the antitumor activity of small molecule MYC inhibitors (MYCi361 and MYCi975), resulting in suppressed tumor growth and tumor sensitization to immune checkpoint inhibition in vivo [46]. For these reasons, *MYC* should be further investigated in the evolution of this disease, which may refine the therapeutic strategies for CCSA. 

For the first time, we detected recurrent alterations using WES in CCSA, discovering mutations in *SRGAP3* (p.D48E, p.K442R and p.R864W) and *KMT2D* (p.Q791E, p.G1218R, p.Q3607_Q3612dup and p.Q3918_Q3920del) in 17% of the analyzed cases as the most common alterations. These mutations, apart from *KMT2D* p.Q791E, were bioinformatically predicted as damaging events. The product of *SRGAP3* has been identified as having a tumor-suppressive role through the negative regulation of RAC [47], suggesting the presence of a dysregulated Rho GTPase pathway in CCSA. *KMT2D* transcript plays an important role in histone methyltransferase [48]; the detection of recurrent *KMT2D* mutations may implicate epigenetic dysregulation in the disease biology of CCSA. Although the functionality of these mutations has not been fully clarified, mutations in *SRGAP3* and *KMT2D* have been detected and considered relevant in a variety of sarcomas, including angiosarcoma, endometrial stromal sarcoma and alveolar rhabdomyosarcoma [49,50,51,52,53]. The investigation of these genes in CCSA is warranted and can be carried out through functional experiments (e.g., loss of function bioassay). Furthermore, compared to previously described activating *MET* mutations located in the tyrosine kinase domain [54], the *MET* mutations (p.S548L) detected in our cohort occurred at the PSI domain that is responsible for positioning the binding site between the ligand and receptor [55]. The biological impact and clinical relevance of mutations in this domain are still unknown but are computationally predicted as nondamaging, implying that the *MET* mutation we detected is unlikely to be an activating mutation and irrelevant for the response to crizotinib.

To broaden the scope of molecular findings, we applied a pathway enrichment analysis to systematically explore mutated CGCs, hopefully identifying overrepresented pathways that are disrupted in CCSA. Our findings indicated that CCSA-associated alterations might be actively involved in transcriptional regulation, SUMOylation, RTK signaling and chromatin organization and modification, as well as MMR. It is not surprising that some of these pathways have already been related to CCSA, such as transcriptional regulation and RTK signaling [8,10]. Furthermore, MMR-related protein MSH6 has previously been tested and was found to be absent in two out of 9 (22%) CCSA cases (one of which showed microsatellite instability) [56,57]. In our cohort, MMR dysregulation was observed in 6 of 24 (25%), which is in-line with the literature and indicates a potential contribution of MMR in a subset of CCSA cases. A further investigation of the tumor mutational burden should be considered to confirm this hypothesis. Additionally, we compared the immunohistochemical findings in MET signaling with the genomic alterations enriched in RTK-related pathways in CCSA. In the cases without MET activation, the RTK-related pathways were found disrupted in all but one case, indicating that genomic alterations may serve as alternative mechanisms that contribute to the activation of MET-downstream molecules in a ligand-independent manner. For instance, the *TSC2* mutations that were detected in three cases in our cohort were known to result in mTOR activation [58]. However, we cannot exclude the presence of other molecular mechanisms (e.g., epigenetic, transcriptional and translational regulation) that could regulate MET signaling. To sum up, we discovered disease-associated pathways and paved the way for further exploration of the pathogenesis of CCSA.

In the correlative analysis, we identified a number of associations between the molecular findings and clinical features (e.g., disease status, OS and PFS). The histopathological correlations revealed that a high-level expression of activated MAPK was significantly associated with a better OS, suggesting that MAPK is a favorable predictive marker for crizotinib treatment. However, we should not overestimate the significance of this finding because of the very small sample size. Next, the copy number gains of chromosomes 1q, 7p and 7q (arm level) were seen more frequently in metastatic CCSAs as compared to the primary tumors, suggesting their potential roles in disease progression, which is also consistent with literature, revealing the association between copy number changes and disease recurrence in various human cancers [59,60]. Recent findings have discovered novel oncogenes on chromosome 7 (e.g., *GTF2IRD1* and *PEG10*) associated with progression in other cancer types [61,62]. The survival analysis highlighted that the loss of 9q and 12q24.33 was associated with poor survival, while chromatin organization deficiency was correlated with longer PFS. As previously reported, the loss of 12q24.33 is an independent predictor of poor PFS in dedifferentiated liposarcoma [63]. Alterations such as *KMT2D* deficiency in chromatin organization have been identified as a favorable prognostic marker in small cell lung cancer, and its potential role to enhance the activity of antitumor treatment in pancreatic adenocarcinoma has been shown [64,65]. To the best of our knowledge, we report here for the first time the potential biomarkers with predictive values for CCSA patients.

There are clear limitations in our study. Due to the lack of germline samples, the genetic analysis was performed based on computational strategies and applications of public databases (e.g., COSMIC and Reactome), which required further experimental validation to confirm the functional consequences of the described alterations. Secondly, these databases have been validated and extensively used, but molecular findings and interactive pathways are still being accumulated. Updates should be considered if more comprehensive genome-wide analysis data are available in the future. Furthermore, for the identification of relevant alterations, we focused on cancer-related genes (CGCs) and involved pathways that were affected by recurrent alterations, which might have missed the importance of rare or noninterpreted alterations. Finally, because the number of assessable cases was too small to perform more advanced statistical analyses, we should not overestimate the statistical power of the described putative biomarkers. Ideally, the observed correlations should be confirmed by treating and analyzing a validation cohort of additional patients, but due to the orphan characteristics of CCSA, this is difficult to achieve. Nevertheless, our study presents one of the biggest subtype-specific series in this orphan disease. The compilation of existing and newly acquired datasets, that is currently planned, will likely broaden our knowledge of CCSA biology.

## 5. Conclusions

We performed a comprehensive histopathological evaluation, characterized the MET-signaling pathway and described the genomic alteration landscape in CCSA and were able to illustrate the molecular heterogeneity of this rare malignancy. We correlated the molecular findings with the outcomes of patients receiving an experimental therapy and identified potential biomarkers with predictive values. Our study provides an insight into the biology of this ultra-rare cancer and will hopefully lead to the identification of novel targets to improve the clinical management of this commonly fatal malignancy.

## Figures and Tables

**Table 1:** CCSA patient characteristics, treatment outcome and availability of the archival tumor tissue used in this exploratory study.

Study SeqID	Gender/ Age (Years)	Origin of Tested Material	MET Status (EORTC 90101 Protocol)		Best Response (RECIST)	Progression Status on Crizotinib	PFS (Months)	Survival Status	OS (Months)	Exploratory Study
			Status	FISH (% Positive Cells)						
5	M/28	P	MET+	nd	not reached	Progression	0.7	Dead	0.7	IHC
12	F/55	P	MET+	70	SD	Progression	2.6	Dead	2.6	IHC + Seq
13	M/33	P	MET−	10	PD	Progression	2.2	Dead	12.1	IHC + Seq
20	M/38	Meta	MET+	90	PD	Progression	1.4	Dead	4.4	Seq
21	M/22	P	MET+	61	PD	Progression	1.1	Dead	2.1	IHC
23	M/55	P	MET+	nd	SD	Progression	2.8	Dead	14.5	IHC + Seq
25	M/29	Meta	MET+	71	SD	Progression	4.3	Dead	7.6	IHC
32	M/44	P	MET+	59	SD	Progression	4.3	Dead	7.6	IHC + Seq
35	M/52	Meta	MET+	71	SD	Progression	9.0	Dead	10.8	IHC + Seq
36	M/29	Meta	MET+	85	SD	Progression	8.3	Dead	21.7	IHC + Seq
49	F/23	Meta	MET+	71	PD	Progression	0.8	Dead	1.4	IHC
52	M/40	P	MET+	40	PD	Progression	0.8	Dead	4.4	IHC + Seq
57	F/38	Meta	MET+	86	SD	Progression	4.8	Alive	14.5	IHC + Seq
60	F/54	P	MET+	73	not reached	Progression	9.2	Dead	9.2	IHC + Seq
68	M/44	P	MET+	87	SD	Progression	5.5	Dead	16.8	IHC + Seq
69	M/43	P	MET-	0	SD	Progression	4.2	Alive	20.7	IHC + Seq
72	F/41	Meta	MET+	85	SD	Progression	2.7	Dead	8.5	IHC
81	M/32	P	MET+	81	SD	Progression	11.3	Dead	11.3	IHC
91	M/57	P	MET+	23	PD	Progression	1.4	Dead	7.6	IHC
101	M/17	P	MET+	31	not reached	Progression	1.5	Dead	1.5	IHC + Seq
106	nd	P	MET+	75	not treated	nd	nd	Alive	nd	IHC + Seq
112	F/56	P	MET+	78	PR	No progression	30.6	Alive	30.6	IHC + Seq
115	F/30	P	MET+	92	SD	No progression	26.3	Alive	26.3	IHC + Seq
121	M/33	Meta	MET+	96	SD	Progression	7.7	Dead	14.0	IHC + Seq
123	M/50	P	MET+	84	SD	Progression	9.6	Dead	15.4	IHC + Seq
124	F/31	P	nd	nd	not reached	Progression	0.8	Dead	0.8	IHC
126	F/47	Meta	MET+	67	SD	Progression	2.8	Dead	8.1	IHC + Seq
127	nd	P	MET+	68	PD	Progression	1.4	Alive	1.4	Seq
135	M/50	P	MET+	73	SD	Progression	5.1	Dead	9.4	IHC + Seq
141	F/49	Meta	MET+	72	not reached	Progression	2.4	Dead	2.4	IHC + Seq
144	F/28	*n*/a	MET+	60	not reached	Progression	0.9	Dead	0.9	IHC
145	M/62	Meta	MET+	34	PD	Progression	1.6	Dead	8.0	IHC
146	M/56	Meta	MET+	71	SD	Progression	9.1	Dead	9.1	IHC + Seq
147	nd	P	MET−	0	not treated	nd	nd	Alive	nd	IHC + Seq

+: positive, −: negative, F: female, FISH: fluorescent in situ hybridization, IHC: immunohistochemistry, M: male, Meta: metastatic lesion, nd: no data, OS: overall survival, P: primary tumor, PD: progressive disease, PFS: progression-free survival, PR: partial response, RECIST: Response Evaluation Criteria in Solid Tumors, SD: stable disease and Seq: sequencing.

**Table 2:** List of the most frequent focal copy number alterations with affected cancer-related genes in 24 clear cell sarcomas.

Altered Cytogenetic Band	Region (Start–End)	Genes from Cancer Gene Consensus Set (COSMIC v89)	Q Values *	# Samples (%)
+8q24.21	113375001–142524999	*EXT1*, *MYC*, *RAD21*, *NDRG1*, *FAM135B*, *CSMD3*	0.008	20 (83.3%)
+8q11.23	51325001–55724999	*TCEA1*	0.034	16 (66.7%)
−9p21.3	2825001–28874999	*CDKN2A*, *JAK2*, *MLLT3*, *NFIB*, *PTPRD*, *PSIP1*, *CD274*, *PDCD1LG2*	0.003	15 (62.5%)
−9p21.2	23825001–30424999		0.002	15 (62.5%)
−10q26.3	129875001–135534747	*MGMT, DUX4*	0.119	15 (62.5%)
−11q24.1	114575001–135006516	*CBL*, *DDX6*, *FLI1*, *KCNJ5*, *MLL*, *PAFAH1B2*, *ARHGEF12*, *BCL9L*, *FOXR1*	0.000	10 (41.7%)
+1q32.1	203825001–205524999	*MDM4*	0.061	10 (41.7%)
+12q15	70225001–70674999		0.008	9 (37.5%)
+5q12.1	58075001–59674999		0.201	7 (29.2%)
−12q24.33	70775001–133851895	*ALDH2*, *BCL7A*, *BTG1*, *POLE*, *PTPN11*, *PTPRB*, *CLIP1*, *TBX3*, *HNF1A*, *NCOR2*, *SH2B3*, *SETD1B*, *CHST11*, *ZCCHC8*, *USP44*	0.199	7 (29.2%)
−22q12.2	29775001–31074999	*NF2*	0.015	7 (29.2%)
+12q13.13	51875001–52874999		0.176	6 (25%)
−14q24.3	68175001–107349540	*AKT1*, *HSP90AA1*, *RAD51B*, *TSHR*, *TCL1A*, *TRIP11*, *GOLGA5*, *DICER1*, *BCL11B*	0.194	6 (25%)
+3q29	195775001–198022430	*TFRC*	0.176	4 (16.7%)
+11p15.1	19475001–20224999		0.203	4 (16.7%)
+11q13.2	66325001–67924999		0.203	4 (16.7%)
+2q22.3	145025001–146074999		0.208	2 (8.3%)

* Regions affected by copy number alterations were selected from the GISTC analysis with a *q*-value < 0.25 as the cut-off. #: number.

## Data Availability

The dataset(s) supporting the conclusions of this article is(are) included within the article and available upon reasonable request.

## Supplementary Materials

The following are available online at https://www.mdpi.com/article/10.3390/cancers13236057/s1, Table S1: List of antibodies and conditions for immunohistochemical characterization in the MET-signaling pathway. Table S2: A summary for the evaluability rate and the level of immunoreactivity for the molecules involved in the MET-signaling pathway. Table S3: Significant alteration-enriched pathways in clear cell sarcoma. Figure S1: Representative images of immunohistochemical staining for MET and the related molecules. Figure S2: Comparison between the histopathological and genomic findings in the context of MET and receptor tyrosine kinase signaling.
