# Supplementary material for: Histopathological and Molecular Profiling of Clear Cell Sarcoma and Correlation with Response to Crizotinib: An Exploratory Study Related to EORTC 90101 “CREATE” Trial
Source: Cancers (Basel). 2021 Dec 1;13(23):6057. doi: 10.3390/cancers13236057 (PMC8657105; doi:10.3390/cancers13236057)
Supplement: Supplementary file 1 [file cancers-13-06057-s001.zip › cancers-1431051-supplementary.pdf]

Supplementary Materials: Histopathological and Molecular Profiling of Clear Cell Sarcoma and Correlation with Response to Crizotinib: An Exploratory Study Related to EORTC 90101 "CREATE" Trial

Che-Jui Lee, Elodie Modave, Bram Boeckx, Silvia Stacchiotti, Piotr Rutkowski, Jean-Yves Blay, Maria Debiec-Rychter, Raf Sciot, Diether Lambrechts, Agnieszka Wozniak and Patrick Schöffski

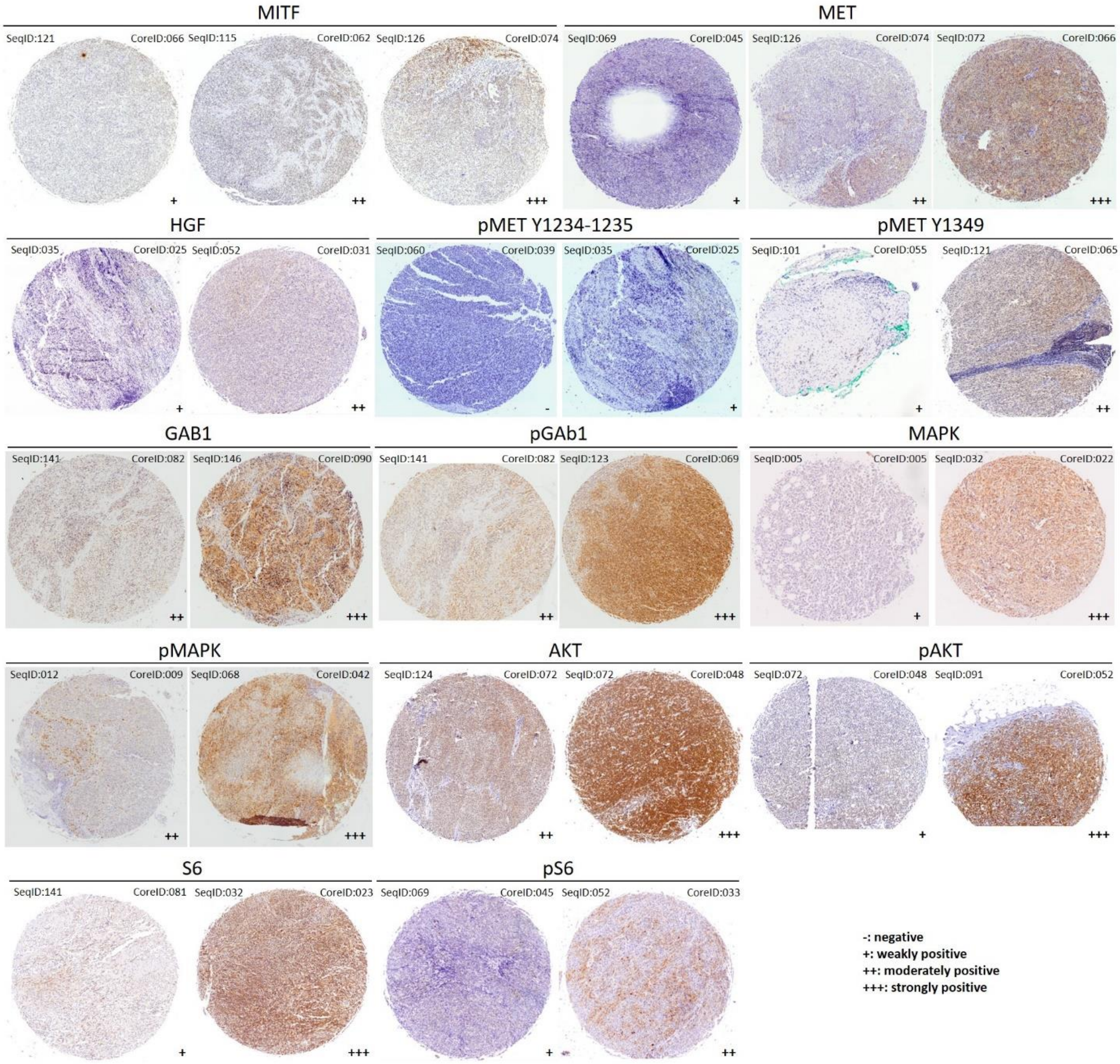

Figure S1. Representative images of immunohistochemical staining for MET and related molecules.

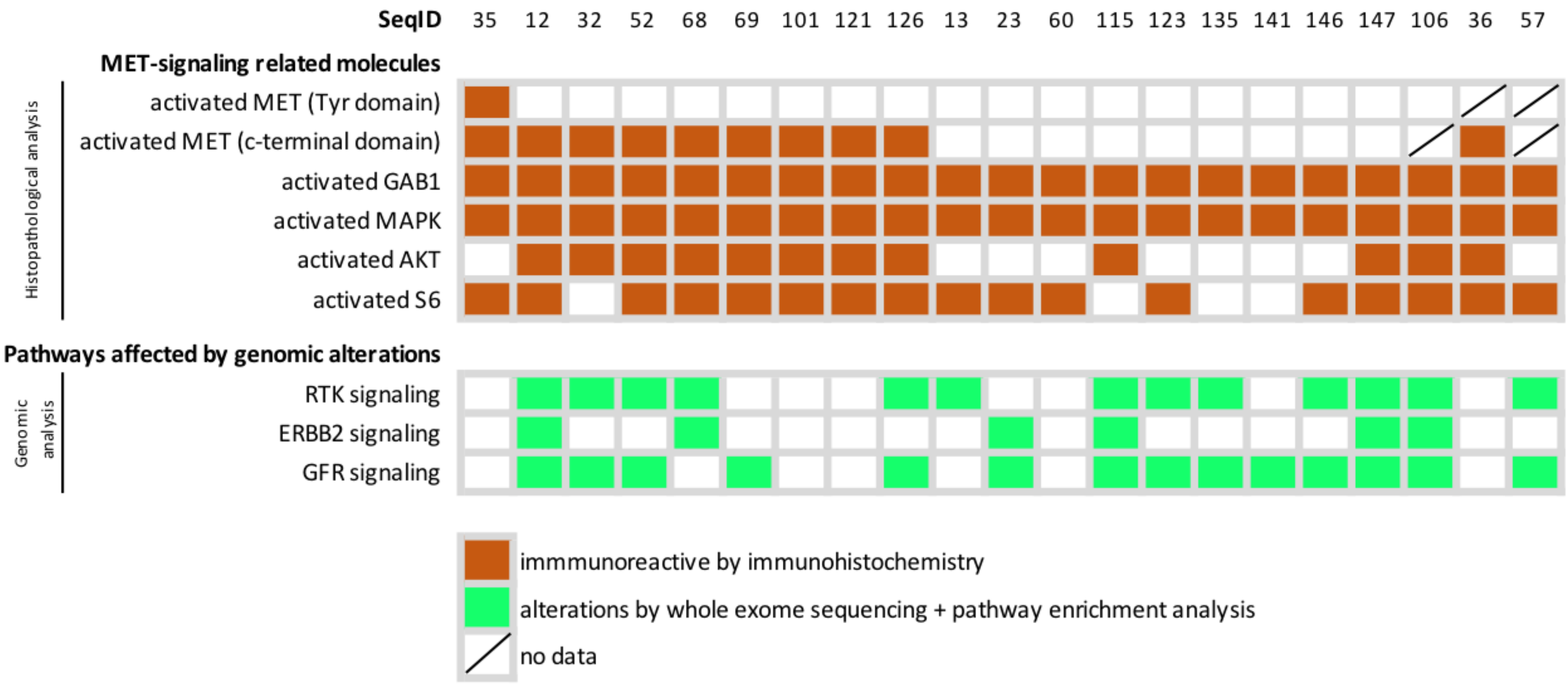

Figure S2. Comparison between histopathological and genomic findings in the context of MET and receptor tyrosine kinase signaling.

Table S1. List of primary antibodies and corresponding coditions for immunohistochemical characterization in MET signaling pathway.

Target	Antibody (Ab) / clone	Company / cat#	Dilution	Incubation temperature / duration
MITF	Anti-MITF rabbit Ab / polyclonal	Sigma-Aldrich / HPA003259	1:500	room temperature / 1 hour
HGF	Anti-HGF rabbit Ab / polyclonal	Sigma-Aldrich / HPA040360	1:50	room temperature / 1 hour
MET	Met XP rabbit Ab / monoclonal (D1C2)	Cell Signaling / 8198	1:100	4 °C / overnight
pMET (Y1234-35)	Phospho-Met (Tyr1234/1235) XP rabbit Ab / monoclonal (D26)	Cell Signaling / 3077	1:25	room temperature / overnight
pMET (Y1349)	Anti-Met (phospho Y1349) rabbit Ab / monoclonal (EP2367Y)	Abcam / ab68141	1:20	room temperature / overnight
GAB1	Anti-GAB1 antibody rabbit Ab / polyclonal	Abcam / ab59362	1:50	4 °C / overnight
pGAB1 (Y659)	Phospho-GAB1 (Tyr659) rabbit Ab / polyclonal	Thermo Fisher Scientific / PA5-38415	1:50	4 °C / overnight
MAPK	p44/42 MAPK (Erk1/2) rabbit Ab / polyclonal	Cell Signaling / 9102	1:100	4 °C / overnight
pMAPK (T202/Y204)	Phospho-p44/42 MAPK (Erk1/2) (Thr202/Tyr204) rabbit Ab / monoclonal (D13.14.4E)	Cell Signaling / 4370	1:200	4 °C / overnight
AKT	Akt (pan) rabbit Ab / monoclonal (C67E7)	Cell Signaling / 4691	1:20	4 °C / overnight
pAKT (S473)	Phospho-Akt (Ser473) rabbit Ab / monoclonal (736E11)	Cell Signaling / 3787	1:20	4 °C / overnight
S6	S6 Ribosomal Protein rabbit Ab / monoclonal (5G10)	Cell Signaling / 2217	1:50	4 °C / overnight
pS6 (S240/244)	Phospho-S6 Ribosomal Protein (Ser240/244) XP rabbit Ab / monoclonal (D68F8)	Cell Signaling / 5364	1:100	room temperature / 30 minutes

MITF: microphthalmia-associated transcription factor, HGF: hepatocyte growth factor, pMET: phosphorylated MET, (p)GAB1: (phosphorylated) GRB2-associated-binding protein 1, (p)MAPK: (phosphorylated) mitogen-activated protein kinase, (p)S6: (phosphorylated) ribosomal S6 kinase.

Table S2. Expression results for molecules involved in MET signaling pathway, assessed in 32 CCSA cases, using tissue microarray.

MET-related molecules	# evaluable cases (%)	Negative (%)	Weakly positive (%)	Moderately positive (%)	Strongly positive (%)
MITF	32 (100%)	7 (22%)	3 (9%)	17 (53%)	5 (16%)
HGF	32 (100%)	26 (84%)	3 (10%)	2 (6%)	0 (0%)
MET	28 (88%)	5 (18%)	5 (18%)	12 (43%)	6 (21%)
pMET (Y1234-35)	28 (88%)	27 (96%)	1 (4%)	0 (0%)	0 (0%)
pMET (Y1349)	28 (88%)	14 (50%)	3 (11%)	10 (36%)	1 (4%)
GAB1	29 (91%)	0 (0%)	1 (3%)	16 (55%)	12 (41%)
pGAB1 (Y695)	30 (94%)	0 (0%)	1 (3%)	12 (40%)	17 (57%)
MAPK	30 (94%)	0 (0%)	2 (7%)	22 (73%)	6 (20%)
pMAPK (T202/Y204)	29 (91%)	6 (21%)	6 (21%)	12 (41%)	5 (17%)
AKT	29 (91%)	1 (3%)	2 (7%)	13 (45%)	13 (45%)
pAKT (S473)	30 (94%)	14 (47%)	11 (37%)	4 (13%)	1 (3%)
S6	29 (91%)	1 (3%)	1 (3%)	5 (17%)	22 (76%)
pS6 (S240/244)	31 (97%)	8 (26%)	5 (16%)	14 (45%)	4 (13%)

MITF: microphthalmia-associated transcription factor, HGF: hepatocyte growth factor, pMET: phosphorylated MET, (p)GAB1: (phosphorylated) GRB2-associated-binding protein 1, (p)MAPK: (phosphorylated) mitogen-activated protein kinase, (p)AKT: (phosphorylated) protein kinase B, (p)S6: (phosphorylated) ribosomal S6 kinase.

Table S3. Significantly alteration enriched pathways in clear cell sarcoma.

Source	Pathways (term_name)	Pathway (term_id)	Adjusted_*p*_value	Intersections
REAC	Gene expression (Transcription)	REAC:R-HSA-74160	9.8E-11	AKT1,AR,ARID2,BLM,BRCA1,CDK12,CDKN2A,CREBBP,DAXX,DICER1,EGFR,FANCC,FAS,FIP1L1,FOXO1,GATA3,GRIN2A,KAT6A,KIT,KMT2C,KMT2D,MAPK1,MDM4,MED12,MET,MLH1,MSH2,NCOR1,NCOR2,NDRG1,NOTCH1,NOTCH2,NUP214,PBRM1,PML,PMS2,RANBP2,RMI2,SF3B1,SMAD3,TAF15,TAL1,TPR,TSC2,WRN,ZFHX3,ZNF521
REAC	Diseases of signal transduction by growth factor receptors and second messengers	REAC:R-HSA-5663202	1.1E-10	AKT1,BCR,BRAF,CREBBP,EGFR,FGFR2,FGFR3,FIP1L1,FOXO1,KDR,KIT,MAP2K1,MAPK1,MET,NCOR1,NCOR2,NF1,NOTCH1,NRAS,NRG1,PDGFRB,PLCG1,SMAD3,TSC2
REAC	Generic Transcription Pathway	REAC:R-HSA-212436	1.3E-09	AKT1,AR,ARID2,BLM,BRCA1,CDK12,CDKN2A,CREBBP,DAXX,EGFR,FANCC,FAS,FOXO1,GATA3,GRIN2A,KAT6A,KIT,KMT2C,KMT2D,MAPK1,MDM4,MED12,MET,MLH1,MSH2,NCOR1,NCOR2,NDRG1,NOTCH1,NOTCH2,PBRM1,PML,PMS2,RMI2,SMAD3,TAF15,TAL1,TSC2,WRN,ZFHX3,ZNF521
REAC	RNA Polymerase II Transcription	REAC:R-HSA-73857	6.4E-09	AKT1,AR,ARID2,BLM,BRCA1,CDK12,CDKN2A,CREBBP,DAXX,EGFR,FANCC,FAS,FIP1L1,FOXO1,GATA3,GRIN2A,KAT6A,KIT,KMT2C,KMT2D,MAPK1,MDM4,MED12,MET,MLH1,MSH2,NCOR1,NCOR2,NDRG1,NOTCH1,NOTCH2,PBRM1,PML,PMS2,RMI2,SMAD3,TAF15,TAL1,TSC2,WRN,ZFHX3,ZNF521
REAC	SUMO E3 ligases SUMOylate target proteins	REAC:R-HSA-3108232	9.0E-09	AR,BLM,BRCA1,CDKN2A,CREBBP,DAXX,FOXL2,NCOA1,NCOR2,NUP214,PML,RANBP2,STAG2,TPR,VHL,WRN
REAC	SUMOylation	REAC:R-HSA-2990846	1.5E-08	AR,BLM,BRCA1,CDKN2A,CREBBP,DAXX,FOXL2,NCOA1,NCOR2,NUP214,PML,RANBP2,STAG2,TPR,VHL,WRN
REAC	Disease	REAC:R-HSA-1643685	6.2E-08	AKT1,BCR,BRAF,CDKN2A,CLTC,CREBBP,DAXX,EGFR,FGFR2,FGFR3,FIP1L1,FOXO1,GNAS,KDR,KIT,MAP2K1,MAPK1,MET,MLH1,MSH2,MSH6,MYH9,NCOR1,NCOR2,NF1,NOTCH1,NOTCH2,NRAS,NRG1,NUP214,PDGFRB,PLCG1,PML,PMS2,RANBP2,RNF213,RPL5,SLC34A2,SMAD3,TAF15,TPR,TSC2
REAC	Transcriptional Regulation by TP53	REAC:R-HSA-3700989	3.8E-07	AKT1,BLM,BRCA1,CDK12,CDKN2A,CREBBP,DAXX,FANCC,FAS,KAT6A,MDM4,MLH1,MSH2,NDRG1,PML,PMS2,RMI2,TAF15,TSC2,WRN
REAC	SUMOylation of DNA damage response and repair proteins	REAC:R-HSA-3108214	2.4E-05	BLM,BRCA1,CDKN2A,NUP214,PML,RANBP2,STAG2,TPR,WRN
REAC	Diseases of Mismatch Repair (MMR)	REAC:R-HSA-5423599	2.5E-05	MLH1,MSH2,MSH6,PMS2
REAC	Regulation of TP53 Activity	REAC:R-HSA-5633007	3.9E-04	AKT1,BLM,BRCA1,CDKN2A,DAXX,KAT6A,MDM4,PML,RMI2,TAF15,WRN
REAC	Diseases of DNA repair	REAC:R-HSA-9675135	6.1E-04	MLH1,MSH2,MSH6,PMS2
REAC	Developmental Biology	REAC:R-HSA-1266738	1.0E-03	AKT1,CACNA1D,CLTC,CLTCL1,CREBBP,DNM2,EBF1,EGFR,EZR,FOXO1,KMT2C,KMT2D,MAP2K1,MAPK1,MED12,MET,MYH9,MYOD1,NCOA1,NCOR1,NCOR2,NOTCH1,NRAS,PLCG1,PTPRC,RPL5,SMAD3,SRGAP3,TAL1
REAC	Signaling by Receptor Tyrosine Kinases	REAC:R-HSA-9006934	1.3E-03	AKT1,BRAF,CLTC,DNM2,EGFR,KDR,KIT,MAP2K1,MAPK1,MET,NCOR1,NRAS,NRG1,NTRK3,PDGFRB,PLCG1,SH2B3,USP8
REAC	PI3K/AKT Signaling in Cancer	REAC:R-HSA-2219528	1.8E-03	AKT1,EGFR,FOXO1,KIT,MET,NRG1,PDGFRB,TSC2
REAC	Chromatin modifying enzymes	REAC:R-HSA-3247509	2.4E-03	ARID2,CREBBP,KAT6A,KMT2C,KMT2D,NCOA1,NCOR1,NCOR2,NSD1,PBRM1,PRDM16,SETD2,TRRAP
REAC	Chromatin organization	REAC:R-HSA-4839726	2.4E-03	ARID2,CREBBP,KAT6A,KMT2C,KMT2D,NCOA1,NCOR1,NCOR2,NSD1,PBRM1,PRDM16,SETD2,TRRAP
REAC	Signaling by ERBB2	REAC:R-HSA-1227986	3.4E-03	AKT1,EGFR,NRAS,NRG1,PLCG1,USP8
REAC	Notch-HLH transcription pathway	REAC:R-HSA-350054	4.2E-03	CREBBP,NCOR1,NCOR2,NOTCH1,NOTCH2
REAC	Mismatch repair (MMR) directed by MSH2:MSH6 (MutSalpha)	REAC:R-HSA-5358565	4.7E-03	MLH1,MSH2,MSH6,PMS2
REAC	Mismatch Repair	REAC:R-HSA-5358508	6.3E-03	MLH1,MSH2,MSH6,PMS2
REAC	Negative feedback regulation of MAPK pathway	REAC:R-HSA-5674499	9.6E-03	BRAF,MAP2K1,MAPK1
